# Supplementary material for: Evaluating situational judgment test use and diversity in admissions at a southern US medical school
Source: PLoS One. 2023 Feb 13;18(2):e0280205. doi: 10.1371/journal.pone.0280205 (PMC9925012; doi:10.1371/journal.pone.0280205)
Supplement: Attachment — Submitted filename: plos1CASPer_ReviewResponseR2.docx [file pone.0280205.s002.docx]

PONE-D-22-17727R1

Evaluating situational judgment test use and diversity in admissions at a southern US medical school

PLOS ONE

Dear Al-Worafi,

Thank you for the opportunity to provide further edits to the manuscript.

We have included the following:

- A rebuttal letter that responds to each point raised by the academic editor and reviewer(s). You should upload this letter as a separate file labeled 'Response to Reviewers'.
- A marked-up copy of your manuscript that highlights changes made to the original version. You should upload this as a separate file labeled 'Revised Manuscript with Track Changes'.
- An unmarked version of your revised paper without tracked changes. You should upload this as a separate file labeled 'Manuscript'.

**Response to Reviewers**

Reviewer #2: The authors have appropriately addressed all reviewers’ comments. The manuscript is ready for publication.

>>Thank you.

Reviewer #3: Dear authors,

Thank you for addressing the previous reviews. In addition to the current revision, would it be possible to provide further information regarding CASper instrument? You mentioned that the reliability of the item is 0.66. It will be useful for the readers if you can describe further what topics/subjects covered by CASper, as well as provide the example of the items. If this has been reported elsewhere, of course the authors may also cite the report. I suggest this to be elaborated in the method section, hence the authors might be able to refer this in the discussion section. This study highlights that the CASper cannot be used as the selection instrument, despite the initial intention. I wonder what's the implication of this, whether the authors would be able to do further actions on the items or not?

>>Thank you for this comment. We have added additional information in the introduction as well as Methods related to CASPer. Since it is a proprietary product, we would not have any ability to influence the items in the instrument. We did comment more on the fact that this instrument could be considered part of holistic review of materials, but that institutions need to do a good job of continuous quality improvement to ensure that they understand the scores for their context.

Reviewer #4: This manuscript reads more to me like a research letter. The authors tried an intervention to specifically broaden selection criteria that might holistically reduce racial/ethnic bias, but it’s unclear that there was any rationale that this tool might do that. If anything the authors could reframe their original hypothesis and manuscript in this manner, i.e. saying that there is no evidence to suggest it would however they sought to explore whether it would and it did not seem to. And perhaps even does the opposite in perpetuating biases.

>>We appreciate your comments. Unfortunately, little has been published about the fact that many medical schools are using CASPer now and consider it to be unbiased. NY Medical College reports these data, but others have not. Anecdotally, we thought it would provide a more unbiased measure based on the data provided by CASPer. That is the reason we wanted to explore the results and thus asked the questions we did.

Intro:
Since CASPER is new to this audience, could use a little more background in the Intro as to how it was validated. Has it previously shown bias or better been shown to not perpetuate bias and inequities in diverse samples?

>>Thank you for this comment. Where CASPer is concerned, no such reports to our knowledge exist. There are other types of situational judgment tests that have shown bias toward ethnic groups, but not in medicine. We discuss that in the Discussion.

Was NHPI analyzed with Asian or with UIM. It should be with UIM. It’s unclear in the Methods and Results as it seems to be state both ways.

>>NHPI was not part of “Asian” and this was corrected in the manuscript.

I’d suggest formal Stats review.

>>We have an experienced statistician on our team who reviewed materials again.

Do the CASPER results differ by race when controlling for other quantitative metrics like GPA or MCAT score?

>>We did not incorporate GPA and MCAT in this analysis. The reason being is that those are primarily considered knowledge-based outcomes. CASPer is meant for non-cognitive abilities and therefore using interview evaluation metrics was more appropriate for an analysis.

Discussion:
The Discussion in general is way too short and superficial / skimpy.

>>We have added to the Discussion.

What did the NYU study show? Should clarify in more detail in the Discussion itself.

>>We have added to the Discussion.

Paragraph 2 gets to the crux of my initial question. If CASPER was introduced as a tool to mitigate bias, increase diversity, and enhance holistic review, where is the evidence that it actually does that? Especially if there are subjective components to the scoring which would be at risk of implicit and explicit biases.

>>We’ve further discussed some of this as part of the expanded discussion. As we note throughout, situational judgment tests are being used in business and medical school admissions as a means of mitigating bias. However, few people have reported findings like ours. Why? We don’t know. But pointing out how different these results are based on UIM status is worth reporting because there may be issues with the grading that CASPer does. As a private business, we have no control over their process.

“applying Methods from”. This statement should be similarly elaborated. What unique recommendations did they have?

>>We have revised this.
